# Facial Reconstruction After Total Resection of Long-Standing Cutaneous Squamous Cell Carcinoma

**DOI:** 10.7759/cureus.71864

**Published:** 2024-10-19

**Authors:** Cristina Lizbeth Puntos-Guízar, Eduardo Yitzhak Muciño-Mondragón, Pablo Belmont-Nava, Brian Bernal-Alferes, Guillermo Sergio Dorantes-Millan

**Affiliations:** 1 Internal Medicine, Centro Médico Nacional 20 de Noviembre, Mexico City, MEX; 2 Dermatology, Centro Médico Nacional 20 de Noviembre, Mexico City, MEX; 3 Surgery, Hospital General Dr. Fernando Quiroz Gutiérrez, Mexico City, MEX

**Keywords:** complex surgical resection, cutaneous squamous cell carcinoma (cscc), dermatology, facial reconstructive surgery, skin disease, surgical dermatology

## Abstract

Cutaneous squamous cell carcinoma (cSCC) is the second most common cutaneous non-melanoma cancer in Mexico, accounting for 20% of all skin cancers. It is considered a global public health concern due to the rising incidence and prevalence, which is associated with an aging population. Treatment for cSCC depends on the severity of the disease, which determines different therapeutic approaches. Reconstructive surgery, as a treatment option, has seen significant advancements over the years, offering new opportunities to improve the quality of life for cSCC patients. Modern surgical techniques require both an aesthetic focus and effective reconstruction to meet the desires and expectations of patients. We present the case of a patient with long-standing SCC located in a facial area that is difficult to access for surgical resection and reconstruction, presenting a therapeutic challenge while achieving an aesthetically harmonious scar.

## Introduction

Cutaneous squamous cell carcinoma (cSCC) is the second most common type of non-melanoma skin cancer (NMSC) in Mexico, representing 20% of all skin cancers [1]. The incidence and prevalence of cSCC are expected to rise among the elderly population, and it should be considered an important diagnosis in this age group. Due to this increased incidence and risk of recurrence, cSCC is regarded as a global public health problem [1].

cSCC oncogenesis is driven by various factors, including environmental, genetic, and epigenetic phenomena. Chronic, cumulative, and lack of photoprotection is the primary environmental factor. Other risk factors contributing to cSCC are age, fair skin, and immunosuppression. [2]. UV radiation contributes to skin cancer by multiple mechanisms: it causes DNA damage, leading to mutations in proto-oncogenes and tumor suppressor genes. This results in cutaneous immune suppression, which weakens immune surveillance and enables unchecked cellular proliferation. In 60% of cutaneous SCC cases, Tp53 mutations are proven by biopsy and molecular biology techniques [3]. In most cases, UV radiation-induced DNA damage is repaired via Tp53. Still, other oncogene mutations persist and accumulate, awaiting a "double-hit" alteration, where tumor suppressor genes undergo loss-of-function mutations that permit cancer to arise [2].

Photoexposed areas, such as the distal upper extremities and the face, are common target regions for SCC manifestation. The clinical heterogeneity of cSCC symptoms makes diagnosis challenging, requiring thorough clinical examination, dermatoscopic exploration, confocal microscopy, and imaging studies such as CT and MRI.

Treatment for cSCC depends on the severity of the condition, guiding distinct treatment lines. Reconstructive surgery has experienced significant advances over the years, improving the quality of life for SCC patients. Demographic characteristics in Mexican population stresses the need to adapt and enhance surgical approach to satisfy the specific needs of heterogeneous groups of cSCC patients. Novel surgical techniques not only focus on reconstruction but also integrate aesthetic considerations, addressing patients' desires and expectations [4]. This article presents a case of long-standing cSCC in a difficult-to-access facial region, posing both a therapeutic and reconstruction challenge.

## Case presentation

A 77-year-old female, a native and resident of Campeche, with incomplete primary schooling, married, evangelical, and a housewife. She has no hereditary or family history of cancer. The patient had a five-year history of systemic arterial hypertension, well-controlled, and a cystectomy 30 years ago. She had an incisional hernia repair four years ago. No history of fractures or transfusions. The patient reported chronic alcoholism for 40 years, drinking to intoxication every three days.

**Figure 1:**
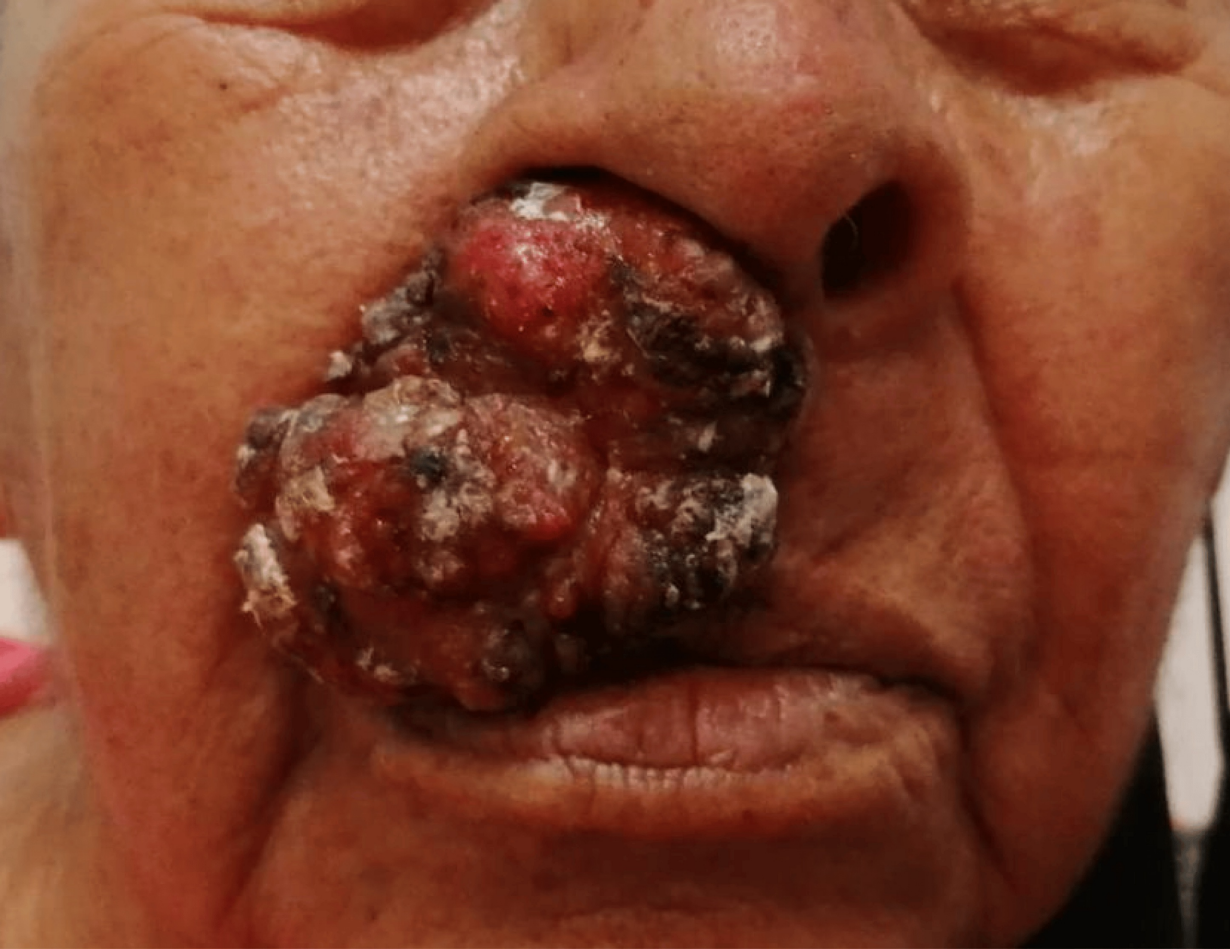
The image shows an exophytic neoformation measuring 8 cm x 6 cm with an irregular surface, keratotic, ulcerated and with crusts on its surface, with a chronic and painful evolution of four years.

Her illness began four years before the evaluation with a lesion 1 cm above her upper lip, which progressively increased in size. She arrived at the hospital with a dermatological tumor affecting the lateral subunit of the upper lip, sparing the nasal mucosa. The lesion was an exophytic neoplasm measuring 8 cm x 6 cm with an irregular surface, keratosis, ulceration, and crusting (Figure 1).

**Figure 2:**
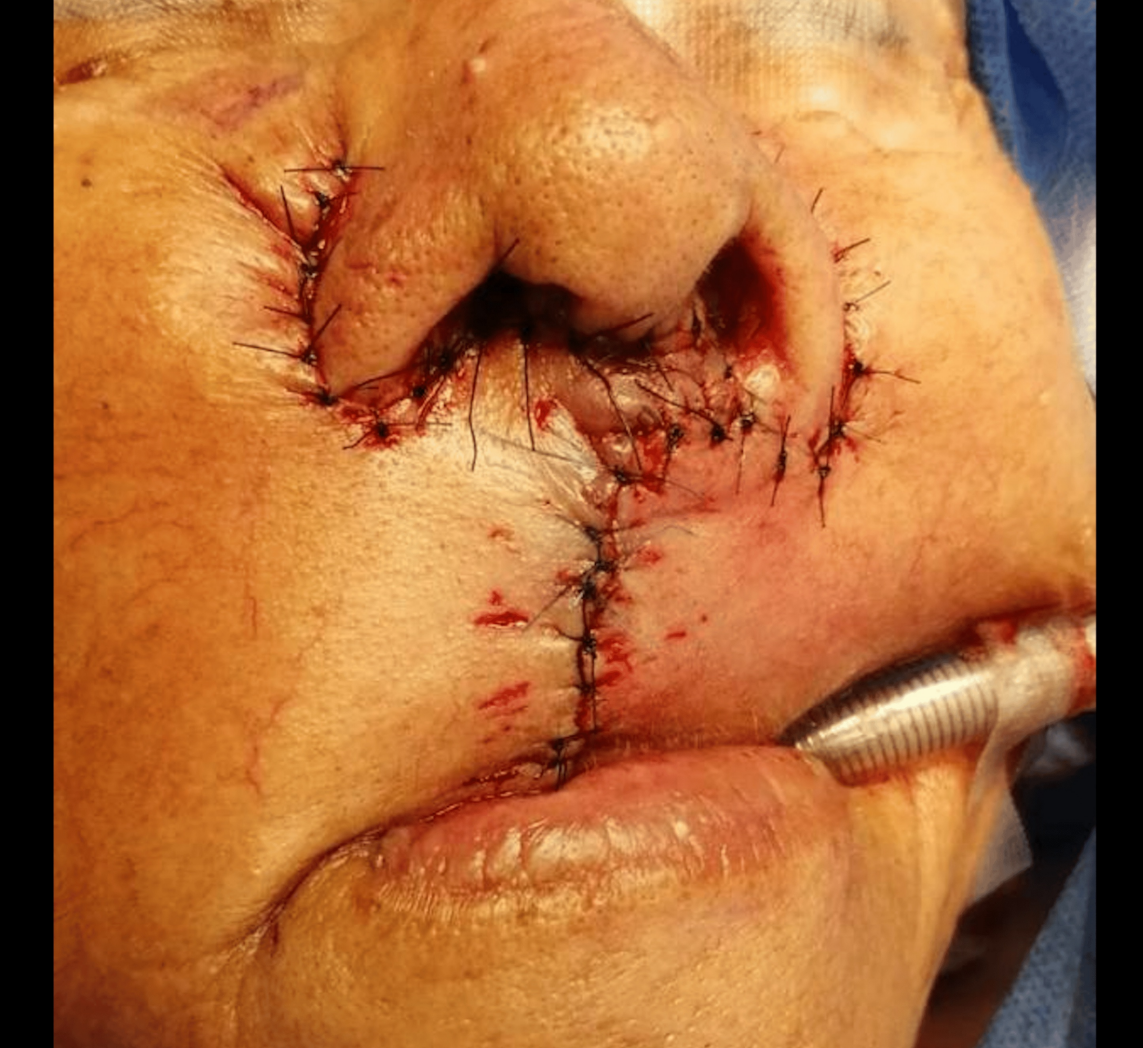
Post-surgical wound. Reconstruction was done with mucosal facing 4-0 vicryl and skin facing with 4-0 nylon, surgical closure.

**Figure 3:**
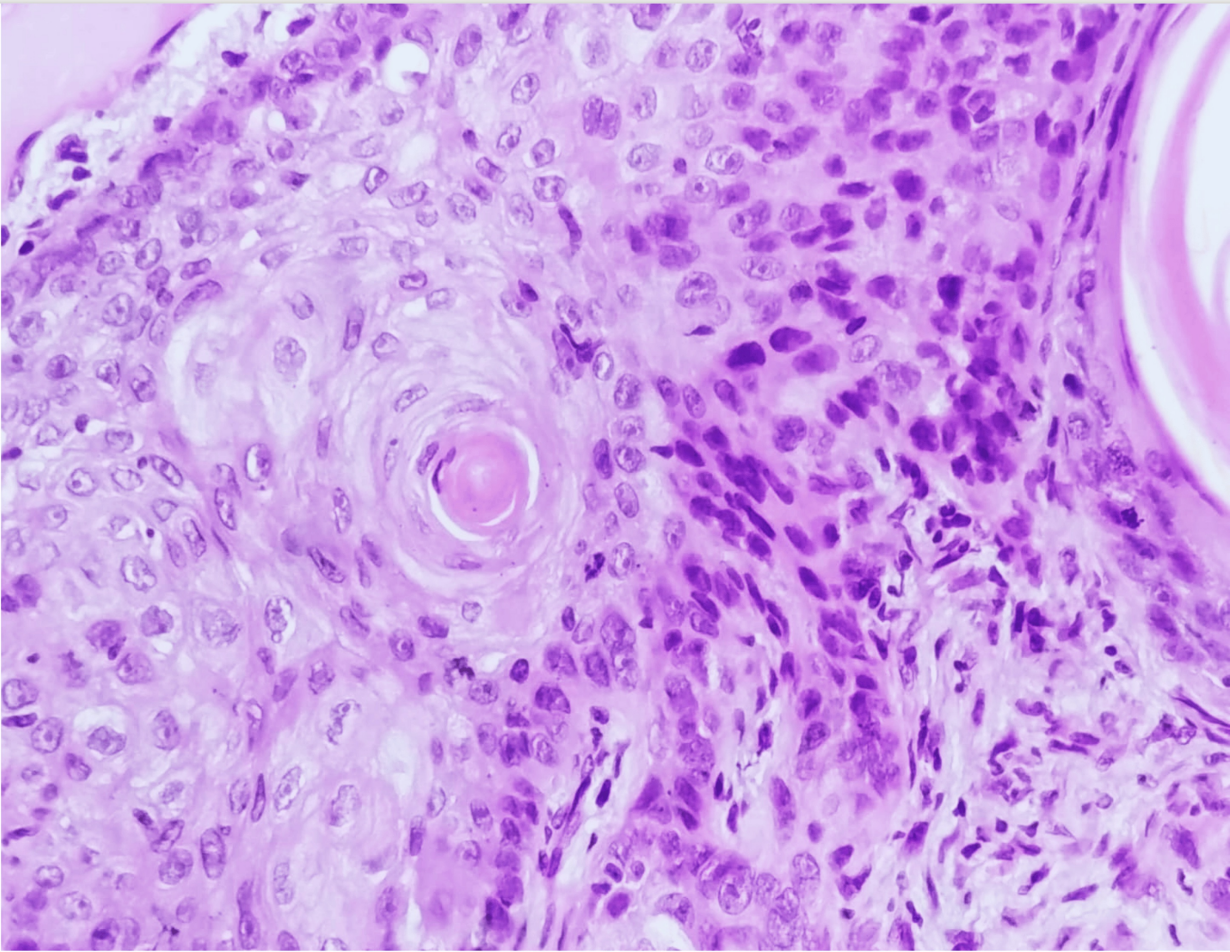
. Haematoxylin-eosin (x400). Atypia characterized by areas of loss of polarity, nuclear irregularities, foci with hyperchromasia. Keratinisation foci and mitotic figures can also be identified.

An incision with a 3 mm margin was made, with a layered approach and dissection of the orbicularis muscle. Both nasal alae were removed, followed by dissection to the oral cavity mucosa. Afterward, 1.5 cm diameter flaps were lifted subcutaneously. Reconstruction was done with mucosal facing using 4-0 Vicryl and the skin with 4-0 Nylon. Hemostasis and airtightness were secured, with no bleeding observed (Figure 2). Intraoperative findings described a 4x3 cm tumor in the upper lip region, with no macroscopic involvement of the oral cavity mucosa, though partial upper lip and right commissure involvement. Margins included 6 mm superior, 4 mm lateral, <1 mm medial, and <1 mm bed, corresponding to the oral cavity mucosa. A biopsy revealed invasive well-differentiated squamous cell carcinoma with ulceration, invading at least the papillary dermis. No lymphovascular or perineural invasion was identified (Figure 3).

**Figure 4:**
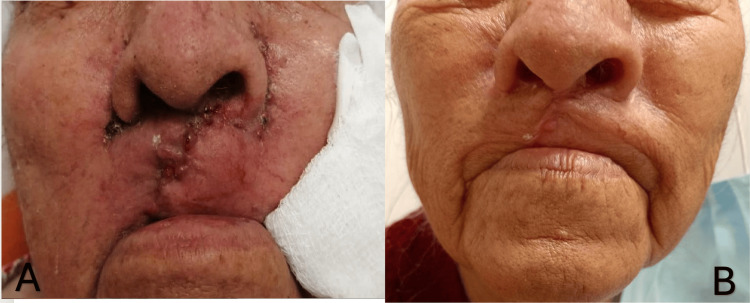
Follow-up three months later, with secondary scar with erythematous borders (A). Follow-up six months later showed a secondary scar lesion 6 cm high on the right side and 2 mm wide (B).

Post-surgery, the patient had no complications, and healing progressed adequately. At a six-month follow-up, she presented a scar measuring 6 cm high and 2 mm wide on the right side (Figure 4).

## Discussion

cSCC is one of the most common skin cancers, with an increasing incidence globally and in Mexico [5]. This rise reflects global trends noted by Leiter et al. (2020), who highlighted that the incidence of skin cancer has surged in recent years due to environmental and lifestyle factors. In our setting, we observe an increasing number of patients presenting with advanced cSCC lesions, as was the case discussed in this article. Many patients delay seeking medical care, worsening their prognosis.

Recurrence rates in locally advanced disease are estimated to be between 40% and 60%, with most recurrences occurring within two years of diagnosis [6]. This underscores the importance of intensive surveillance during this period. Pathological evaluation of surgical specimens is crucial, as factors like tumor size, growth pattern, and invasion depth influence staging and the decision to apply adjuvant treatments [5].

The diagnosis of squamous cell carcinoma (SCC) begins with a detailed clinical examination. In this case, the patient presented with a large lesion, which justified the need for additional imaging studies, such as computed tomography (CT) and magnetic resonance imaging (MRI), to assess the possible involvement of deep structures [5].

A biopsy is essential to confirm the diagnosis. In our patient, an excisional biopsy was performed to obtain sufficient tissue for analysis. Various biopsy techniques are available, such as punch or incision biopsies, depending on the location and extent of the tumor [6]. In addition, in high-risk patients, an evaluation of regional lymph nodes is critical to detect metastases. Techniques such as sentinel lymph node biopsy or advanced imaging studies can be used to achieve this [5].

The primary treatment for cSCC is surgery. In this case, due to the extent of the tumor, a complete surgical resection with wide margins was performed to reduce the risk of recurrence [6]. Mohs surgery is a technique that offers greater precision, particularly in cases where the preservation of healthy tissue is paramount. This technique has been shown to lower recurrence rates compared to conventional surgery [2].

For patients with locally advanced or metastatic tumors, radiotherapy is an effective therapeutic option when surgery is not feasible [6]. In cases of advanced cSCC, immunotherapy with agents such as cemiplimab or vaccinating a patient with its tumor mutations has shown promising results by improving patient survival [2,7].

Postoperative facial reconstruction is essential for patients with extensive cSCC, such as the one presented in this clinical case. Skin grafting and local flap techniques were used to repair defects resulting from surgical resection, enabling improved functional and aesthetic restoration [6]. Adequate reconstruction also facilitates postoperative surveillance, allowing for the early detection of potential recurrences [5].

The psychological impact on patients undergoing extensive facial resections should not be underestimated. Providing integrated support, including physical rehabilitation and psychological counseling, is key to helping patients adapt to changes in their appearance and maintain a satisfactory quality of life [5].

Postoperative reconstruction not only improves the patient's aesthetic and functional outcomes but also assists in clinical follow-up by enabling easier detection of potential recurrences. In conclusion, a multidisciplinary approach is essential to optimize outcomes in patients with advanced cSCC and enhance their quality of life [2,5,6].

## Conclusions

The comprehensive management of SCC and nasosinus tumors remains complex due to the varied clinical presentations. Emphasis should be placed on recognizing risk factors such as chronic alcoholism and making timely diagnoses based on clinical features in conjunction with pathological anatomy. Surgical management, including resection and reconstruction, is an essential factor in determining patient prognosis. In this case, the tumor’s anatomical location and extent posed a significant challenge for an appropriate surgical approach, particularly in the midfacial region, given the complexity of the involved structures. Efforts were made to achieve aesthetically harmonious scarring, considering the emotional impact of visible scars in an exposed area, which helped the patient feel confident about the aesthetic outcomes. Early detection of these tumors remains essential to improve clinical and surgical outcomes.

Moreover, SCC tumors require a holistic approach and evaluation through clinical examination, dermatoscopy, rhinoscopy, and complementary imaging studies such as CT or MRI to ensure adequate and timely intervention. Postoperative follow-up is crucial, focusing on assessing recurrences and monitoring complications during the healing and recovery period.
